# Rapid Estimation of Myelin for Diagnostic Imaging and Quantification of Therapy Responses in Multiple Sclerosis

**DOI:** 10.1111/jon.70128

**Published:** 2026-02-13

**Authors:** Evangelos Katsarogiannis, Russell Ouellette, Johan Virhammar, Anne‐Marie Landtblom, Joachim Burman, Tobias Granberg, Shala G. Berntsson

**Affiliations:** ^1^ Department of Medical Sciences, Neurology Uppsala University Uppsala Sweden; ^2^ Department of Clinical Neurosciences Karolinska Institutet Stockholm Sweden; ^3^ Department of Neuroradiology Karolinska University Hospital Stockholm Sweden

**Keywords:** aHSCT, multiple sclerosis, myelin, rituximab, therapy response

## Abstract

**Background and Purpose:**

Recent MRI developments have allowed for in vivo myelin imaging in clinically feasible time frames. This retrospective study aimed to evaluate the ability of the Rapid Estimation of Myelin for Diagnostic Imaging (REMyDI) technique in monitoring longitudinal myelin changes and brain atrophy in persons with multiple sclerosis (pwMS) undergoing treatment with rituximab or autologous hematopoietic stem cell transplantation (aHSCT).

**Methods:**

Between May 2017 and January 2022, 62 pwMS treated with either rituximab (*n* = 25) or aHSCT (*n* = 37) underwent brain MRI scans at three time points. A 3 Tesla brain MRI was performed, including 3D T1‐weighted imaging, 3D T2‐weighted fluid‐attenuated inversion recovery imaging, and 2D multi‐dynamic multi‐echo imaging for REMyDI and brain volumetrics. Longitudinal changes in imaging parameters and associations with the Expanded Disability Status Scale and Symbol Digit Modalities Test were analyzed using mixed‐effects models.

**Results:**

The rituximab group exhibited increases in whole‐brain myelin (+0.25 mL per year), cortical myelin (+0.11 mL per year), and myelin in normal‐appearing deep gray matter (NADGM) (+0.02 mL per year). In contrast, these measures were stable or declined in the aHSCT group. Brain parenchymal fraction showed a larger reduction in the rituximab group (−0.68% per year) compared to the aHSCT group (−0.24% per year). Myelin‐related imaging measures showed positive but nonsignificant associations with clinical parameters.

**Conclusions:**

REMyDI enables longitudinal assessment of myelin‐related metrics in vivo, which complements conventional brain volumetrics and is suitable for monitoring treatment responses in MS.

## 1 Introduction

Multiple sclerosis (MS) is the most prevalent immune‐mediated demyelinating disease affecting the central nervous system (CNS). A hallmark of MS pathogenesis is inflammation in the white and gray matter, characterized by immune cell infiltration with focal and diffuse demyelination [1, 2]. A critical aspect involves cytotoxic T cells, which induce oligodendrocyte cell death and thereby impair the synthesis and repair of the myelin sheath. Although remyelination may occur over time, oligodendrocytes often fail to restore the myelin sheath effectively [1, 2]. Demyelination is a crucial factor in both physical and cognitive decline in persons with MS (pwMS) [3, 4].

Rituximab, the most commonly used disease‐modifying treatment (DMT) for relapsing‐remitting MS (RRMS) in Sweden, has recently been proven to effectively prevent clinical and radiological relapses in a Phase 3 study [5]. In 2023, rituximab was included on the World Health Organization model list of essential medicines for MS [6]. Autologous hematopoietic stem cell transplantation (aHSCT) is another therapy that has been increasingly used in recent years. aHSCT aims to reset the immune system by eliminating autoreactive lymphocytes and inducing long‐term remission [7]. In a 2021 meta‐analysis, the 5‐year relapse‐free survival in MS treated with aHSCT was 81% [8]. Cerebrospinal biomarkers of myelin injury commonly return to normal values in the years following aHSCT, suggesting that demyelinating processes may be halted by aHSCT [9]. Longitudinal magnetization transfer imaging studies in patients treated with aHSCT have shown that demyelination and remyelination within MS lesions follow heterogeneous and prolonged trajectories, with ongoing myelin loss and repair detectable months to years after lesion formation [10, 11].

The potential of remyelination as a marker of effective immunomodulation in MS has been limited by the sparsity of clinically applicable methods for myelin estimation. Although several myelin imaging techniques exist for research, they often require offline post‐processing and lack clinical application [12]. The evaluation of myelin content and brain atrophy in MS has significantly advanced with the advent of sophisticated imaging techniques. Rapid Estimation of Myelin for Diagnostic Imaging (REMyDI) is a recently clinically approved myelin imaging technique validated ex vivo and in vivo in MS and healthy controls [13]. REMyDI has proven sensitive to both focal and diffuse demyelination, correlating with clinical disability measures such as the Expanded Disability Status Scale (EDSS) and Symbol Digit Modalities Test (SDMT) [13, 14]. Notably, the capacity of REMyDI to detect short‐term treatment‐induced myelin changes and remyelination in the CNS has been recently demonstrated in a conceptually important study of a rare treatable primary demyelinating disorder (methylenetetrahydrofolate reductase deficiency) [15]. However, REMyDI has yet to be applied to study treatment effects on myelin content in MS.

This study aimed to assess the utility of the REMyDI technique in monitoring myelin changes and brain atrophy during high‐efficacy MS treatment in two independent treatment groups: one with rituximab and the other with aHSCT. In addition, we investigated the longitudinal correlation between radiological and clinical parameters in patients undergoing these treatments.

## 2 Methods

### 2.1 Ethical Considerations

This study was approved by the Regional Ethics Board of Uppsala (approval number 2016/203). All patients provided informed and written consent following the Declaration of Helsinki.

### 2.2 Study Population and Clinical Data

The patients included were identified at the Department of Neurology of Uppsala University Hospital, Uppsala, Sweden, between May 2017 and January 2022. Demographic and clinical data on age, gender, disease duration, previous DMTs, and EDSS and SDMT scores were retrieved from the Swedish MS registry and patient charts. A summary of the study cohort demographics is presented in Table 1.

**TABLE 1:** Participant demography and clinical characteristics.

Characteristic	All participants	Rituximab	aHSCT
Participants, *n* (%)	62	25 (40%)	37 (60%)
Baseline scan	38 (61%)	23 (92%)	15 (41%)
Scan 1	59 (95%)	24 (96%)	35 (95%)
Scan 2	28 (45%)	12 (48%)	16 (43%)
Females, *n* (%)	45 (73%)	19 (76%)	26 (70%)
Age, years	32.3 ± 8.8	35.8 ± 9.7	30 ± 7.5
Disease duration, years	5.5 ± 5.5	4.7 ± 4.7	6.0 ± 6.0
Previous DMTs, median (IQR)	1.0 (1.0–2.0)	1.0 (0.0–1.0)	1.5 (1.0–3.0)
Time since treatment start, months			
Baseline scan	−2.8 ± 3.1	−3.0 ± 3.4	−2.4 ± 2.7
Scan 1	14.8 ± 7.7	11.6 ± 7.3	16.9 ± 7.2
Scan 2	21.7 ± 6.0	21.2 ± 5.5	22.2 ± 6.4
EDSS scores, median (IQR)			
Baseline	2.0 (1.0–2.5)	1.5 (1.0–2.0)	2.0 (1.5–3.5)
Scan 1	1.5 (1.0–3.0)	1.5 (0.5–2.5)	2.0 (1.0–3.0)
Scan 2	1.8 (0.0–2.3)	2.0 (1.3–2.3)	1.5 (0.0–2.3)
SDMT scores			
Baseline	51.7 ± 12	51.4 ± 8.3	52.0 ± 15.4
Scan 1	52.4 ± 9.2	52.1 ± 8.1	52.6 ± 9.9
Scan 2	52.8 ± 14.5	55.5 ± 5.6	51.6 ± 17.2

*Note*: All values are given as mean ± standard deviation unless otherwise specified. IQR indicates the interquartile range (25th–75th percentile).

Abbreviations: aHSCT, autologous hematopoietic stem cell transplantation; DMTs, disease‐modifying treatments; EDSS, Expanded Disability Status Scale; IQR, interquartile range; *n*, number of participants; SDMT, Symbol Digit Modalities Test.

The inclusion criteria for the study were:

- Diagnosis of MS: Participants should have a confirmed diagnosis of MS according to the 2011 or 2017 McDonald criteria.
- Age: 18–65 years of age at enrollment.
- Treatment: Ongoing treatment with rituximab or previous aHSCT, regardless of the number of previous DMTs or disease duration.
- Clinical parameters (EDSS and SDMT data) were available at baseline and posttreatment.

Baseline MRI scans were available only for a subset of patients, and both the number and timing of follow‐up examinations varied substantially. Because a few scans occurred far outside the expected clinical follow‐up period and represented clear temporal outliers, and given the generally heterogeneous spacing of examinations, imaging analyses were restricted to MRI studies performed within 1000 days after treatment initiation. This approach ensured temporal comparability across participants while allowing inclusion of all patients who had at least one REMyDI scan within this interval.

Because REMyDI was introduced into the clinical protocol during the study period, the available MRI examinations varied not only in number but also in completeness, with some scans—due to technical problems—generating quantifiable measurements for only a subset of imaging variables. As a result, not all patients had multiple posttreatment scans suitable for full radiological analysis. Within the 1000‐day window, 3 rituximab‐treated patients and 16 aHSCT‐treated patients contributed only a single complete REMyDI scan. These individuals were nevertheless retained, as the longitudinal modeling framework accommodates unbalanced and partially observed data, and each scan—whether isolated or part of a series—provides valid information for estimating the relationship between imaging metrics and time since treatment initiation.

### 2.3 Treatment Description

In the aHSCT group, autologous hematopoietic stem cells were mobilized with a single dose of 2 g/m^2^ cyclophosphamide followed by filgrastim 5–10 µg/kg/day for 6–7 days and then harvested approximately 10 days after the start of the mobilization regimen. No ex vivo graft manipulation was performed. Patients were conditioned with cyclophosphamide (200 mg/kg) and rabbit antithymocyte globulin (6 mg/kg). Prophylaxis for fungal, viral, and bacterial infection was administered during neutropenia. Prophylaxis for herpes viruses and *Pneumocystis jirovecii* continued for 3 months.

In the rituximab group, treatment started with an initial intravenous infusion of 1000 mg rituximab, followed by 500 mg every 6 months.

### 2.4 Image Acquisition

Brain MRI was performed on a maximum of three occasions (a pretreatment baseline scan, Scan 1, and Scan 2) on a single Philips Ingenia Elition X 3 Tesla scanner with a standard clinical head‐neck coil to acquire three sequences: (i) 3D T1‐weighted imaging; (ii) 3D T2‐weighted fluid‐attenuated inversion recovery (FLAIR) imaging; and (iii) 2D multi‐dynamic multi‐echo (MDME) imaging. The high‐resolution T1‐weighted imaging was obtained using both 3D turbo spin echo (TSE) and 3D gradient‐recalled echo (GRE) sequences in the axial plane. The 3D TSE T1‐weighted sequence used a field of view (FOV) of 240–250 mm, in‐plane resolution of 0.4–0.9 mm, slice thickness of 1.0–1.1 mm, repetition time (TR) of 400–600 ms, echo time (TE) of 20–29 ms, and a flip angle of 90°. The 3D GRE sequence was acquired with an FOV of 240 mm, in‐plane resolution of 0.9 mm, slice thickness of 1.0 mm, TR of 9 ms, TE of 4 ms, and a flip angle of 8°. The T2‐weighted FLAIR imaging was performed using a 3D TSE sequence in the sagittal plane with an FOV of 240–250 mm, in‐plane resolution of 0.5–0.7 mm, slice thickness of 1.0–1.1 mm, TR of 4800 ms, TE of 313–340 ms, inversion time of 1650 ms, and a flip angle of 90°. An MDME TSE sequence was acquired in the axial plane using a 2D acquisition with a FOV of 230 mm, in‐plane resolution of 0.45 mm, slice thickness of 4.0 mm, TR of 4680 ms, and dual TE of 12.5 and 100 ms. The inversion time was non‐fixed, and a flip angle of 90° was used.

### 2.5 Imaging Post‐Processing

The 3D T1‐weighted and FLAIR imaging were postprocessed using the longitudinal Sequence Adaptive Multimodal SEGmentation (SAMSEG) pipeline in FreeSurfer (v. 7.3.2, Harvard University, Boston, MA, USA) to obtain segmentations of lesions, normal‐appearing white matter (NAWM), and normal‐appearing deep gray matter (NADGM) and cortex. The resulting segmentations were reviewed to screen for gross segmentation failures, none of which were identified; therefore, no edits were made to maintain consistency between the two timepoints.

The 2D MDME imaging was processed with SyMRI (v. 0.56.13, Synthetic MR, Linköping, Sweden) to obtain the REMyDI myelin value. The intracranial volume segmentation from SyMRI, which is very robust [16, 17], was used to normalize the volumetric measurements from FreeSurfer. A synthetic 2D T1‐weighted image was registered to the 3D T1‐weighted image in the space of the longitudinal FreeSurfer SAMSEG segmentation. Following this registration, the resulting registration matrix was copied to align the REMyDI myelin imaging sequence into the neuroanatomical volume space. This approach is facilitated by the synthetic T1 and the REMyDI myelin map being derived from the same MDME sequence and, therefore, inherently sharing the same imaging space. The myelin content was extracted from the respective tissue compartments using FSL tools [18]. Lesions were enumerated automatically using the FSL‐cluster tool applied to the SAMSEG longitudinal lesion segmentation mask [18].

The resulting imaging measures were the brain parenchymal fraction (BPF), lesion count, lesion volume, NAWM fraction, NADGM fraction, whole‐brain myelin value, lesion myelin value, NAWM myelin value, NADGM myelin value, and cortical myelin value.

### 2.6 Statistical Analysis

The normality of data was tested by histogram analysis, ensuring that the skewness and kurtosis values were within a range of ± 1. We used the *X* ^2 ^test (2‐tailed) to assess sex differences between the two treatment groups.

We used linear mixed‐effects models, with intercept as a random factor by the participant, to explore how brain atrophy rate and myelin value change over time after treatment with aHSCT and rituximab. This statistical model accounts for within‐subject correlations over time. The dependent variables in the models were brain atrophy, rate or myelin value change, and separate models were fitted for each outcome. The fixed effects included in the models were treatment, time since treatment, and the interaction between the two factors. To investigate the potential associations of EDSS and SDMT with brain atrophy rate and myelin value change, the fixed effects included in the mixed models were treatment, time since treatment, EDSS, and SDMT. Bonferroni–Holm correction was applied to adjust for multiple testing in analyses of EDSS and SDMT.

### 2.7 Sensitivity Analysis

To check the robustness of the results of the primary data analyses, we chose to perform sensitivity analyses. This was due to the study's retrospective nature, lack of baseline data in the aHSCT group compared to the rituximab group (41% vs. 92%), outliers, and different time intervals between scans. For the sensitivity analyses, we focused on the subgroups within each treatment group that had access to scans before treatment initiation. Sensitivity analyses were performed using the Wilcoxon signed‐rank test to compare myelin metrics and BPF at baseline with the last posttreatment scan for each patient. A *p* value of < 0.05 was considered significant. Analyses were performed using SAS 9.4 software (SAS Institute).

## 3 Results

### 3.1 Clinical Characteristics and Demographics

A total of 62 patients were included in the study, 25 (40%) were treated with rituximab and 37 (60%) underwent aHSCT. Table 1 summarizes the demographic and clinical characteristics. Out of all patients, 61% (38/62) were examined at baseline, 95% (59/62) at Scan 1, and 45% (28/62) at Scan 2. The average age was higher in the rituximab group (*p* = 0.014), while disease duration tended to be longer in the aHSCT group, (*p* = 0.29). The median number of previous DMTs was higher in the aHSCT group compared to the rituximab group (*p* < 0.001). The EDSS and SDMT scores remained rather stable across the different time points.

### 3.2 Longitudinal Imaging Results in the Rituximab Cohort

#### 3.2.1 Mixed‐Effects Model Analysis

**FIGURE 1:**
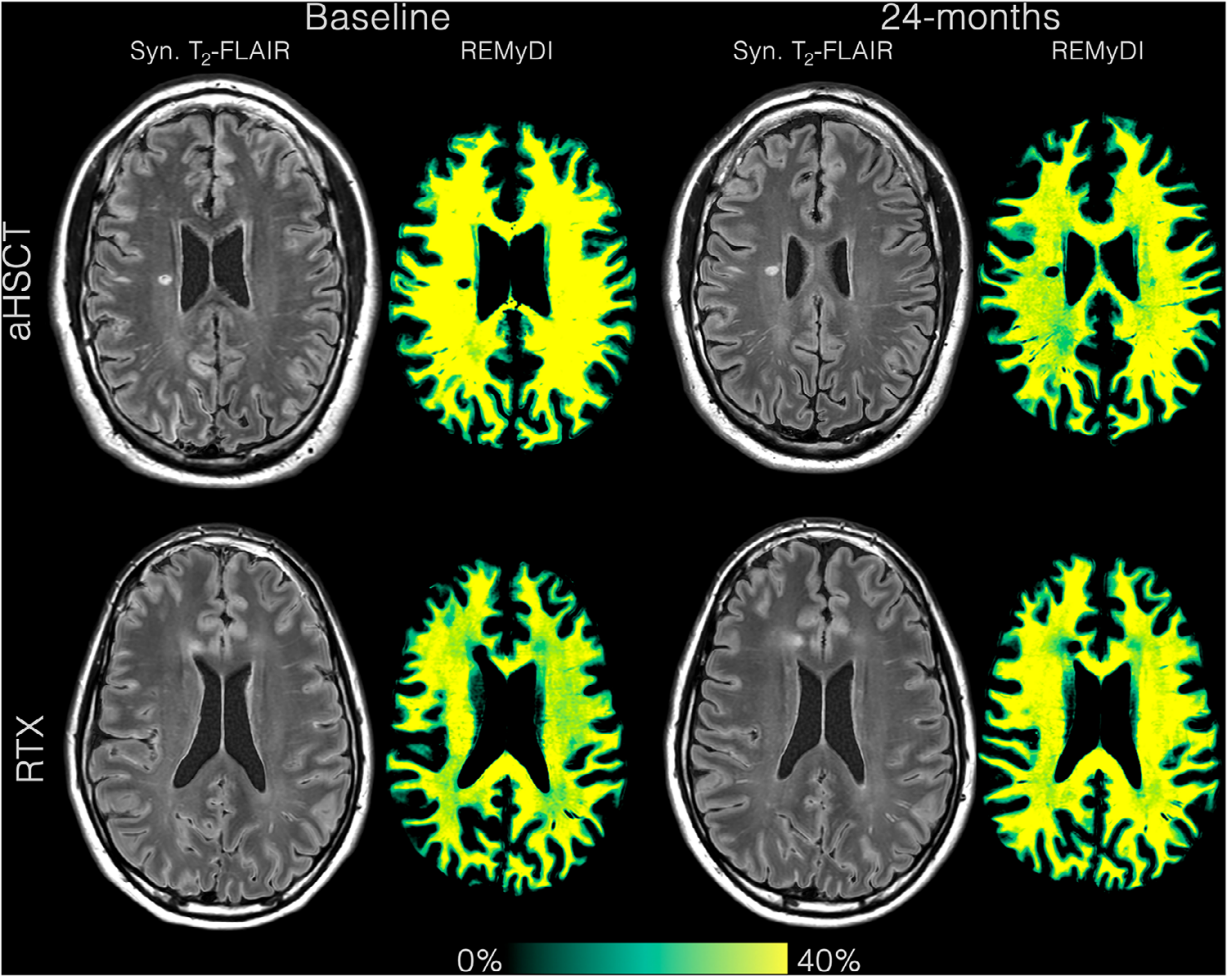
Baseline versus follow‐up T2‐fluid‐attenuated inversion recovery and Rapid Estimation of Myelin for Diagnostic Imaging (REMyDI) acquisitions in two representative study participants. A 32‐year‐old female participant with 12 years of disease duration who underwent autologous hematopoietic stem cell transplantation (aHSCT) is shown at baseline and 24‐month follow‐up, and a 40‐year‐old male participant with 1.5 years of disease duration treated with rituximab (RTX) is shown at baseline and 24‐month follow‐up.

Representative conventional MRI (T2‐FLAIR) and REMyDI‐derived myelin maps from a patient treated with rituximab at baseline and follow‐up are shown in Figure 1. The results from the mixed‐effects models are summarized in Table 2. In the rituximab‐treated cohort, whole‐brain myelin increased over time at an estimated rate of 0.25 mL per year (95% CI: 0.04–0.45). Cortical myelin also increased, with an annual change of 0.11 mL (95% CI: 0.00–0.22). NAWM myelin demonstrated a modest increase of 0.19 mL per year (95% CI: 0.00–0.38), while NADGM myelin increased by 0.02 mL per year (95% CI: 0.01–0.04). Lesion myelin showed a small annual increase of 0.02 mL (95% CI: −0.00 to 0.04).

**TABLE 2:** Estimated slopes (change per year) from linear mixed‐effects models.

	Rituximab number of observations/patients	Rituximab change/year (95% CI)	aHSCT number of observations/patients	aHSCT change/year (95% CI)
BPF	52/23	−0.68 (−1.04, −0.31)	66/37	−0.24 (−0.61, 0.13)
Lesion count	52/22	−0.14 (−0.35, 0.07)	49/25	−0.03 (−0.24, 0.19)
Lesion volume	49/21	−1.69 (−8.34, 4.95)	51/26	−0.07 (−6.59, 6.46)
NADGM fraction	44/19	0.08 (−0.01, 0.17)	45/24	−0.05 (−0.14, 0.03)
NAWM fraction	44/19	−0.19 (−0.66, 0.29)	45/24	0.76 (0.32, 1.21)
Cortical myelin	44/19	0.11 (0.00, 0.22)	45/24	−0.11 (−0.21, ‐0.01)
Lesion myelin	44/ 19	0.02 (−0.00, 0.04)	46/25	0.01 (−0.01, 0.03)
NADGM myelin	44/19	0.02 (0.01, 0.04)	45/24	−0.00 (−0.02, 0.01)
NAWM myelin	44/19	0.19 (0.00, 0.38)	45/24	0.20 (0.02, 0.38)
Whole myelin	52/23	0.25 (0.04, 0.45)	66/37	−0.05 (−0.25, 0.16)

*Note*: For each imaging variable, the table reports the number of patients and the total number of observations included in the analysis. A square root transformation was applied to lesion count, lesion myelin, and lesion volume.

Abbreviations: BPF, brain parenchymal fraction; CI, confidence interval; NADGM, normal‐appearing deep gray matter; NAWM, myelin‐related metrics across normal‐appearing white matter.

Among the remaining imaging variables, BPF declined over time. Lesion count showed a decreasing trend, whereas lesion volume remained stable. NAWM fraction decreased longitudinally, while the NADGM fraction exhibited a slight increase.

#### 3.2.2 Sensitivity Analysis

Sensitivity analyses comparing baseline pretreatment imaging with the last available follow‐up scan largely supported the mixed‐effects model findings. BPF showed a significant reduction from baseline to last follow‐up. All myelin measures demonstrated numerical increases after treatment, although none reached statistical significance in paired testing. Lesion count and lesion volume did not change significantly between baseline and follow‐up. NAWM and NADGM fractions remained stable in the sensitivity analysis, consistent with the modest longitudinal changes observed in the mixed‐effects models (Table 3).

**TABLE 3:** Comparison of baseline and last follow‐up imaging measures for the rituximab group.

Imaging measure	Patients (*N*)	Rituximab baseline	Rituximab last follow‐up	*p* value
Lesion count, *n*, median (IQR)	20	11.0 (7.0–16.0)	9.0 (6.0–16.0)	0.3157
Lesion volume, mL, median (IQR)	19	4.04 (2.16–8.94)	4.58 (1.87–7.35)	0.9843
BPF, %	20	85.96 (4.27)	84.97 (4.68)	0.0002^*^
NAWM fraction, %	17	28.79 (3.25)	28.93 (2.32)	0.8536
NADGM fraction, %	17	2.41 (0.24)	2.50 (0.31)	0.2842
Whole‐brain myelin, mL	20	11.38 (1.37)	11.71 (1.55)	0.1194
NAWM myelin, mL	17	7.86 (1.29)	8.17 (1.11)	0.0714
NADGM myelin, mL	17	0.33 (0.04)	0.35 (0.05)	0.1454
Cortical myelin, mL	17	2.19 (0.32)	2.22 (0.40)	0.7119
Lesion myelin, mL	17	0.22 (0.09)	0.23 (0.11)	0.4038

*Note*: The Wilcoxon signed‐rank test compares the changes from the baseline to the last scan. Values are reported as means with standard deviations unless otherwise specified. IQR indicates the interquartile range (25th–75th percentile). *N* denotes the number of observations. Statistically significant *p* values are indicated by an asterisk (^*^).

Abbreviations: BPF, brain parenchymal fraction; NADGM, normal‐appearing deep gray matter; NAWM, myelin‐related metrics across normal‐appearing white matter.

### 3.3 Longitudinal Imaging Results in the aHSCT Cohort

#### 3.3.1 Mixed‐Effects Model Analysis

Representative conventional MRI (T2‐FLAIR) and REMyDI‐derived myelin maps from a patient treated with aHSCT at baseline and follow‐up are shown in Figure 1. The results from the mixed‐effects models are summarized in Table 2. In the aHSCT cohort, whole‐brain myelin remained stable over time, with an estimated annual change of −0.05 mL (95% CI: −0.25 to 0.16). Cortical myelin declined at a rate of −0.11 mL per year (95% CI: −0.21 to −0.01). NAWM myelin increased by 0.20 mL per year (95% CI: 0.02–0.38), whereas NADGM myelin remained stable at −0.00 mL per year (95% CI: −0.02 to 0.01). Lesion myelin demonstrated a small increase of 0.01 mL per year (95% CI: −0.01 to 0.03).

For the remaining imaging variables, BPF showed a mild decline over time. Lesion count and lesion volume remained stable. NAWM fraction increased longitudinally, while the NADGM fraction decreased.

#### 3.3.2 Sensitivity Analysis

Sensitivity analyses in the aHSCT cohort were largely concordant with the mixed‐effects results. NAWM fraction increased significantly from baseline to last follow‐up, while cortical myelin declined significantly. Whole‐brain myelin, NAWM myelin, NADGM myelin, lesion myelin, lesion count, lesion volume, and BPF did not show significant baseline‐to‐follow‐up changes (Table 4).

**TABLE 4:** Comparison of baseline and last follow‐up imaging measures for the aHSCT group.

Imaging measure	Patients (*N*)	aHSCT baseline	aHSCT last follow‐up	*p* value
Lesion count, *N*, median (IQR)	11	13.0 (4.0–26.0)	13.0 (6.0–16.0)	0.2500
Lesion volume, mL, median (IQR)	11	5.11 (3.99–11.17)	4.86 (3.19–8.38)	0.5771
BPF, %	12	85.34 (5.06)	84.72 (5.16)	0.1406
NAWM fraction, %	10	27.70 (2.75)	28.97 (2.47)	0.0273^*^
NADGM fraction, %	10	2.43 (0.37)	2.25 (0.22)	0.1602
Whole‐brain myelin, mL	12	11.38 (1.70)	11.33 (1.37)	0.7705
NAWM myelin, mL	10	7.48 (1.33)	7.81 (1.27)	0.1602
NADGM myelin, mL	10	0.34 (0.04)	0.33 (0.03)	0.5566
Cortical myelin, mL	10	2.22 (0.30)	1.94 (0.25)	0.0195^*^
Lesion myelin, mL	10	0.22 (0.09)	0.23 (0.10)	0.5566

*Note*: The Wilcoxon signed‐rank test compares the changes from the baseline to the last scan. Values are reported as means with standard deviations unless otherwise specified. IQR indicates the interquartile range (25th–75th percentile). *N* denotes the number of observations. Statistically significant *p* values are indicated by an asterisk (^*^)

Abbreviations: aHSCT, autologous hematopoietic stem cell transplantation; BPF, brain parenchymal fraction; NADGM, normal‐appearing deep gray matter; NAWM, myelin‐related metrics across normal‐appearing white matter.

### 3.4 The Correlation Between Clinical and Radiological Parameters

In linear mixed‐effects models adjusted for treatment, time since treatment initiation, and repeated measures, all myelin‐related imaging measures showed positive associations with EDSS; however, none remained statistically significant after adjustment for multiple testing. With respect to cognitive performance, all myelin metrics except lesion myelin demonstrated positive, but nonsignificant, associations with SDMT *Z* scores, and no radiological parameter showed a statistically significant correlation with SDMT *Z* scores. After Bonferroni–Holm correction, lesion myelin had the lowest *p* value with a corrected *p* value of 0.64, whereas all other analyses had corrected *p* values of 1.

## 4 Discussion

This is the first study to use REMyDI to evaluate potential treatment effects on myelin content in MS. We found that REMyDI is indeed able to capture dynamics of demyelination and remyelination in vivo in MS. This is in line with the results of a previous pivotal study of three persons with methylenetetrahydrofolate reductase deficiency, a very rare primary demyelinating disease, where remyelination was demonstrated following administration of appropriate therapy with oral treatment of betaine, methionine, vitamin B_12_, B_6_, and folic acid [15]. In this study, we thereby corroborate the use of clinically approved myelin imaging as a quantitative biomarker to study treatment effects also in MS. This adds to the current literature that has previously validated REMyDI ex vivo and shown that it correlates to cognitive and physical MS‐related disability [13, 19].

Rituximab treatment was associated with small increases in SyMRI‐derived myelin metrics, suggesting myelin preservation or modest recovery. While a small reduction in lesion number was observed, the longitudinal myelin changes are unlikely to be explained solely by this decrease. Myelin content was assessed separately within lesions, NAWM, NADGM, and cortex, revealing treatment‐related effects across both lesional and non‐lesional tissue. The observed myelin increases could therefore reflect broader microstructural normalization accompanying reduced inflammatory burden, rather than merely resulting from a lower lesion count. Rituximab treatment resulted in an annual increase in NADGM myelin, which may indicate potential effects on myelin within the deep gray matter. These findings align with recent studies showing that most rituximab‐treated patients achieved disease stabilization (no evidence of disease activity, NEDA‐3) and a reduced rate of confirmed disability worsening [20]. Evidence highlighting rituximab's efficacy and favorable safety profile contributed to its growing adoption as a first‐line treatment [5]. This possibly accounts for the shorter disease duration and lower prior use of DMTs in the rituximab group.

The apparent discrepancy between greater BPF decline and relatively stable or increasing myelin in the rituximab group may reflect differences in what these measures capture. While BPF is sensitive to global tissue‐volume changes, SyMRI myelin metrics primarily reflect tissue integrity and relaxation properties, providing complementary information about myelin status. The aHSCT group demonstrated a reduction in myelin content, both in terms of whole‐brain and cortical myelin values, over time. This result could be attributed to the fact that many patients, especially those in the aHSCT group, were enrolled without a baseline examination since REMyDI was not available at the time of their treatment, which could have underestimated the effect of aHSCT on posttreatment measurements. Notably, several measurements in the aHSCT group were conducted more than a year after transplantation. Previous studies have shown that aHSCT displays a substantial portion of its immunomodulatory effects within the first year [21, 22]. The delayed timing of these measurements could fail to capture the early myelin‐preserving benefits of aHSCT. One potential explanation for this delay is that a significant number of follow‐up scans in the aHSCT group were postponed or rescheduled due to the COVID‐19 pandemic. In contrast, a large portion of the rituximab cohort was enrolled in the study after the pandemic.

Another important aspect is that the aHSCT cohort comprised patients with a longer, more severe form of the disease that had been refractory to previous treatments, indicating more advanced stages of MS. Prolonged disease duration in aHSCT is linked to higher posttransplant progression rates, highlighting the impact of advanced baseline characteristics on treatment efficacy [23].

Interpretation of the aHSCT findings can be attributed to several factors. Differences in baseline disease burden, timing of posttreatment imaging, and transient physiological changes during immune reconstitution could all influence myelin and volumetric measures. These possibilities remain hypothetical and should be interpreted with caution.

Furthermore, we observed a small annual decrease in BPF in the aHSCT group. A recent study showed an initial decrease in brain volume, likely due to pseudoatrophy, which in many cases normalized at the 24‐month follow‐up [24]. The same study also showed that posttransplant sNfL levels, especially in patients with RRMS, decreased markedly after 24 months, suggesting reduced inflammation‐related axonal damage [18].

The capacity of aHSCT to achieve long‐term remission has been highlighted in several studies. In one study, 70% of patients reported NEDA over 10 years [25]. Another reported NEDA in 62% of patients for at least 4 years [21]. The annualized relapse rate (ARR) showed a substantial decrease, dropping from 1.2 before aHSCT to 0.1 post‐aHSCT [21]. A reduction in EDSS scores was reported in 79% of patients with MS [25]. Similarly, our study observed a decline in EDSS scores posttreatment in the aHSCT group (Table 1).

Walker et al. (2014) found that while aHSCT initially caused cognitive decline and brain volume reduction in MS patients, cognitive functions mostly returned to baseline by 24 months [26]. We observed stable cognitive outcomes in the aHSCT group despite reduced myelin values.

Several advanced imaging techniques have been developed to measure myelin content in the brain with high repeatability and reproducibility [27, 28]. MRI‐based measures of myelin values showed strong correlations with histopathological findings, indicating their potential utility as noninvasive biomarkers for assessing myelin integrity and remyelination in MS [13, 29]. Pathological studies suggest that disability progression in progressive MS (PMS) can occur without cerebral white matter (WM) demyelination. However, other factors, such as cortical atrophy and neurodegeneration, play significant roles [30].

We found complex relationships between myelin metrics and clinical outcomes in MS patients. Overall, associations between myelin measures and disability or cognitive performance were modest and did not remain statistically significant after correction for multiple testing. While some directional trends were observed, these should be interpreted cautiously given the exploratory nature of the analyses and limited statistical power. Previous studies have associated functional mobility with reduced myelin disruption [4]. Among all radiological parameters, cortical myelin showed the strongest association with cognitive performance measured by SDMT, although this did not reach statistical significance. This is noteworthy as previous studies have revealed significant cortical myelin loss in early‐stage MS [31].

Our study was not designed to compare the effects of rituximab and aHSCT directly. The methodology and design were not optimized for a head‐to‐head evaluation of these treatments. Instead, the study should be viewed as a parallel analysis, where we separately evaluated the impacts of rituximab and aHSCT on myelin metrics and BPF in MS patients.

This study had several limitations. First, the late integration of REMyDI into MRI protocols led to missing baseline data for many patients, particularly in the aHSCT group, resulting in an imbalance between treatment arms. Second, some follow‐up scans were missing due to postponed examinations or technical issues, leading to incomplete longitudinal data. Third, the time intervals between baseline, Scan 1, and Scan 2 were highly variable among patients, which, together with the retrospective design, introduces variability in timing and limits causal inference. Fourth, the relatively small sample size, especially when divided into subgroups, restricted the generalizability of the findings and reduced statistical power to detect significant differences or trends. Fifth, we did not include baseline measures such as lesion load or disease duration as covariates in the mixed‐effects models due to limited observations per participant and missing baseline data, particularly in the aHSCT group. This may have introduced residual confounding, although baseline differences between groups were relatively small and thus unlikely to have had a major impact. Finally, the absence of a healthy or untreated MS control group prevents clear differentiation between treatment‐related effects and natural disease trajectories. Given the exploratory nature of these analyses, the reported correlations should be interpreted with caution.

Our findings underscore the nuanced role of myelin quantification in understanding progression and monitoring treatment responses in MS patients. This study highlights REMyDI´s role as an advanced imaging technique, providing complementary insights to conventional brain volumetrics. Future research should focus on prospective longitudinal studies and larger cohorts to validate our findings and explore the potential role of REMyDI as a clinical biomarker in MS in guiding personalized treatment strategies.

## Funding

Johan Virhammar is funded by the Swedish Society for Medical Research (SG‐22‐0192‐H‐01). Tobias Granberg is funded by Alzheimersfonden, Karolinska Institutet (CIMED junior grant), Region Stockholm (ALF Medicine grants), the Swedish Society for Medical Research (Big grant), and Merck's Grant for Multiple Sclerosis Innovation Award.

## Conflicts of Interest

The authors declare no conflicts of interest.
